# A Maize *Calmodulin-like 3* Gene Positively Regulates Drought Tolerance in Maize and *Arabidopsis*

**DOI:** 10.3390/ijms26031329

**Published:** 2025-02-04

**Authors:** Dan Li, Hanqiao Wang, Fushun Luo, Mingrui Li, Zhiqiang Wu, Meiyi Liu, Zhen Wang, Zhenyuan Zang, Liangyu Jiang

**Affiliations:** College of Agriculture, Jilin Agricultural University, Changchun 130118, China; 15248522089@163.com (D.L.); wanghanqiao02@163.com (H.W.); 17585496641@163.com (F.L.); mqmqlee@163.com (M.L.); 17396774079@163.com (Z.W.); l2932144311@163.com (M.L.); wangzhen021010@163.com (Z.W.)

**Keywords:** calmodulin-like, *ZmCML3*, drought stress, maize

## Abstract

Drought stress is one of the important abiotic stresses that affects maize production. As an important Ca^2+^ sensor, calmodulin-like proteins (CMLs) play key roles in plant growth, development, and stress response, but there are a limited number of studies regarding CMLs in response to drought stress. In this study, a Calmodulin-like gene, namely *ZmCML3*, was isolated from maize (*Zea mays* L.). The coding sequence (CDS) of ZmCML3 was 474 bp and a protein of 158 aa which contains three EF-hand motifs. ZmCML3 was localized within the nucleus and plasma membrane. The expression of *ZmCML3* was induced by polyethylene glycol (PEG) 6000, NaCl, methyl jasmonate (MeJA), and abscisic acid (ABA). Overexpression of *ZmCML3* resulted in enhanced drought tolerance in maize through increasing proline (Pro) content and the activity of peroxide (POD) and superoxide dismutase (SOD). Meanwhile, *ZmCML3* also positively regulated the expression of drought stress-responsive genes in maize under drought stress treatment. Taken together, *ZmCML3* acts as a positive regulator in maize response to drought stress. These results will provide theoretical basis for breeding drought tolerance maize variety.

## 1. Introduction

Drought stress is one of the most dangerous environmental factors [1]. In the past decade, drought stress has resulted in global crop production of USD ~30 billion [2]. Maize, as one of the most important food crops, is being affected by drought stress [3]. Drought stress can seriously affect its growth and lead to 20–50% yield reduction, or even no harvest [4,5]. Therefore, it is of great significance to cultivate new drought tolerance maize varieties through molecular breeding technology.

Plants are often subjected to various abiotic and biotic stresses during their growth process, including pathogen infection, drought, cold, and salt stresses [6,7]. The plant has involved various mechanisms to counteract environmental stresses, including the calcium (Ca^2+^) signaling pathway, MAPK signaling pathway, plant hormone signaling pathway, and reactive oxygen species (ROS) production [8,9]. Ca^2+^, as a second messenger, is an essential nutrient in the plant growth and development process [10,11]. When plants are subjected to various stresses, the concentration of Ca^2+^ rapidly changes, and activates a series of signaling pathway to ensure the Ca^2+^ concentration to drop a low level [12]. The Ca^2+^ signal is divided into three steps: (1) recognition (Ca^2+^ concentration changes), (2) signal relay (Ca^2+^ sensor binds to Ca^2+^ and transmits signals), (3) altered metabolic, including novel gene expression [13,14].

To date, four Ca^2+^ sensors have been identified in plants, including calcium-dependent protein kinase (CDPK), calmodulin (CaM), and calmodulin-like (CML) and calcineurin B-like protein (CBL) [15]. Most Ca^2+^ sensor proteins have a typical helix-loop-helix structure, called EF-hand motif, which is highly conserved and has the ability of Ca^2+^ binding [16,17]. CMLs, as plant specific Ca^2+^ sensors, are involved in plant growth and development and the response to various stresses [18,19]. The *CML* genes in plants have been extensively identified [20]. For example, a total of 52, 80, 230, 32, 144, and 50 *CML* genes have been identified in tomato, barley, wheat, rice, soybean, and *Arabidopsis*, respectively [21,22,23,24,25,26].

An increasing number of studies have demonstrated that the *CML* genes participate in plant growth and development and various stress responses. For example, GbCML45 and GbCML50 can positively regulate *verticillium wilt* resistance through ethylene and salicylic acid (SA) signaling pathways [27]. An overexpression of *CaCML13* in transgenic plants displays enhanced resistance against *Ralstonia solanacearum* infection [28]. Overexpression of MdCML3 can increase the tolerance of apples to salt stress [29]. AhCML69 can improve the resistance of tobacco to *R. solanacearum* infection [30]. AtCML8 can enhance a plant’s resistance against several pathogens, including *R. solanacearum*, *Xanthomonas*, and *Phytophthora* [31]. Overexpression of *MtCML42* leads to enhanced cold stress tolerance in *Medicago truncatula* [32]. *MpCML40* overexpressing *Arabidopsis* displays increased tolerance to salt stress [33]. In maize, ZmCML25 was found to participate in regulating the maize resistance against gray leaf spot [34]. *ZmCML48* and *ZmCML50* can respond to drought stress [35]. However, the roles of CMLs in maize are involved in response to drought stress remain unclear.

Previously, we have identified 46 *CMLs* genes in maize and found that a *CML* gene was significantly upregulated in response to drought stress through transcriptome sequencing [36]. In this study, we cloned the *CML* gene, namely *ZmCML3*. The subcellular localization and expression pattern analysis were used to investigate the role of *ZmCML3*. Meanwhile, we detected the function of *ZmCML3* overexpressing maize and *Arabidopsis* plants in response to drought stress. This study provides a theoretical foundation for revealing the function of *ZmCML3* under drought stress and will be beneficial to breed drought-tolerant maize varieties.

## 2. Results

### 2.1. Cloning and Characterization of ZmCML3 Gene

**Figure 1:**
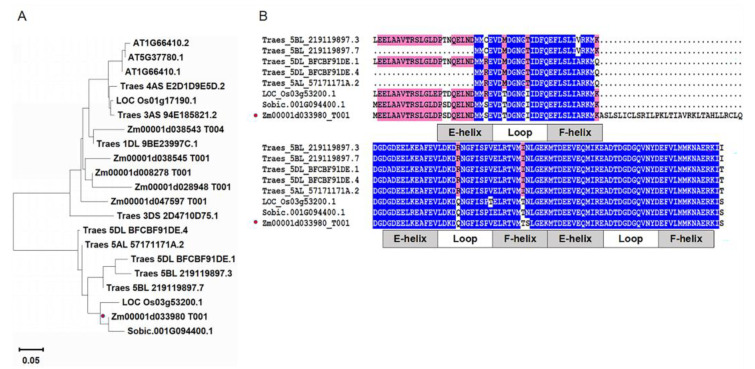
Bioinformatics analysis of ZmCML3. (**A**) Phylogenetic tree of ZmCML3 homologous genes with other plant species. (**B**) The multi-sequence alignment of ZmCML3 orthologous proteins. The different background colors represent the similar degree of amino acid sequences. Blue: the similar degree of amino acid sequences is greater than or equal 75%. Pink: the similar degree of amino acid sequences is less than 75% and greater than or equal 50%. Gray: the similar degree of amino acid sequences is less than 50%. The red dots: Mark ZmCML3 (Zm00001d033980).

The full-length gene (Zm00001d033980) was cloned from maize inbred line B73 and named *ZmCML3*. The coding sequence (CDS) of *ZmCML3* was 474 bp and encoded 158 amino acids with a 17.57 Kda (pI 4.35) molecular mass. The *ZmCML3* gene was located on chromosome 5. Phylogenetic analysis showed that ZmCML3 exhibits high identities with Sobic.001G094400.1 (78%) (Figure 1A). The multi-sequence alignment of ZmCML3 orthologous proteins showed that ZmCML3 contains three conserved EF-hand motifs (Figure 1B). These results indicate that ZmCML3 has similar function with CMLs from other plants.

### 2.2. Analysis of ZmCML3 Cis-Acting Elements

*Cis*-acting elements play a crucial role in regulating gene expression. In order to explore the role of *ZmCML3* involved in stress response, the online database PlantCARE (https://bioinformatics.psb.ugent.be/webtools/plantcare/html/, accessed on 16 July 2024) was used to analyze the *cis*-acting elements of the *ZmCML3* promoters (upstream 2000 bp). As shown in Table S1, the promoter of *ZmCML3* contains Antioxidant Response Elements (ARE), MYB binding site (MBS), ABA responsive element (ABRE), CGTCA-motif, and TGACG-motif. The results indicated that *ZmCML3* may participate in response to various abiotic stresses.

### 2.3. Expression Analysis of ZmCML3 Under Different Stresses

**Figure 2:**
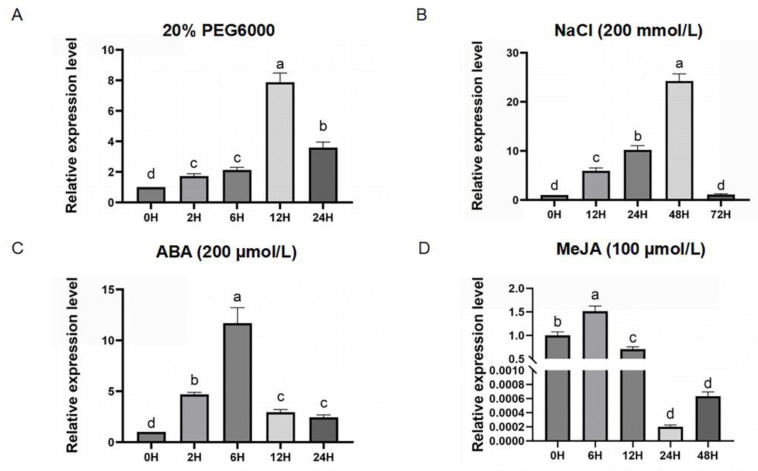
The expression levels of *ZmCML3* in maize under different treatments, including (**A**) PEG6000 (20%), (**B**) NaCl (200 mmol/L), (**C**) ABA (200 µmol/L), and (**D**) MeJA (100 µmol/L). The experiments were performed with three biological replicates. *ZmTub* (GRMZM2G066191) was used as an endogenous reference for data standardization. The significance difference was analyzed using one-way ANOVA according to letter marking method. The different letters indicate significant differences at *p* < 0.05.

Much evidence has shown that the *CML* genes play an important role in response to abiotic stresses [37,38]. In order to investigate whether *ZmCML3* is involved in abiotic stress and the hormonal signaling pathway, the expression levels of *ZmCML3* were detected. Under PEG 6000 (20%) treatment, the expression level of *ZmCML3* gradually increased from 0 h (h) to 6 h and exhibited a peak at 12 h (Figure 2A). Under NaCl (200 mmol/L) treatment, *ZmCML3* expression increased rapidly, and reached a maximum level (6-fold) at 48 h (Figure 2B). *ZmCML3* expression was markedly induced, with the highest expression level of *ZmCML3* was observed at 6 h (25-fold), by exposure to abscisic acid (ABA) (200 µmol/L) (Figure 2C). *ZmCML3* expression rapidly increased at 6 h (1.5-fold), then decreased, reaching its lowest point at 24 h by exposure to methyl jasmonate (MeJA) (100 µmol/L) (Figure 2D). These results indicated that *ZmCML3* was significantly induced by PEG 6000 (20%), NaCl, ABA, and MeJA treatments. PEG 6000 is an important osmotic regulator and can simulate drought stress, thus *ZmCML3* may be involved in drought stress response.

### 2.4. Subcellular Localization of ZmCML3 Protein

**Figure 3:**
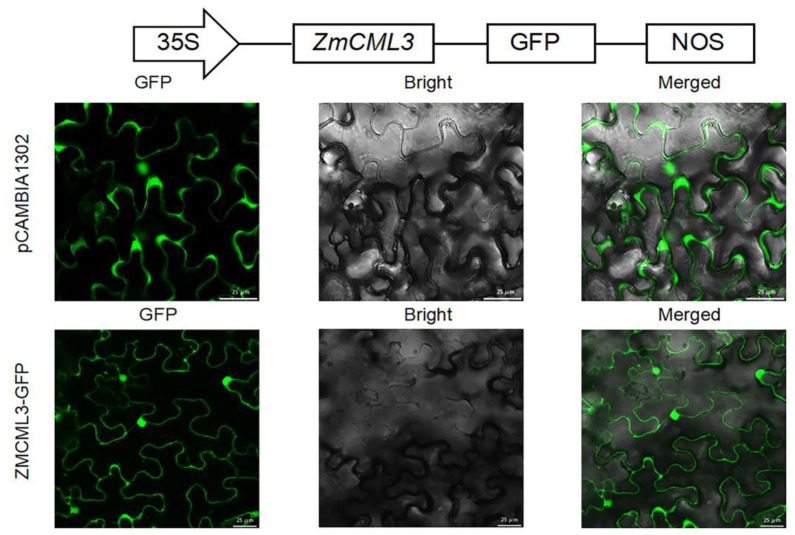
Subcellular localization of ZmCML3 fusion protein. The subcellular localization was observed by confocal laser scanning microscope. Each image has no less than three fields of view. Bar = 25 μm.

In order to investigate the subcellular localization of the ZmCML3 protein, the fusion vector ZmCML3-GFP was generated and transfected into tobacco (*N. benthamiana*) leaf epidermal cells. As shown in Figure 3, the fluorescence signals from both pCAMBIA1302 and the ZmCML3-GFP were mainly observed in the nucleus and plasma membrane. These results showed that ZmCML3 protein is located within the nucleus and plasma membrane.

### 2.5. Overexpression of ZmCML3 Can Enhance Drought Tolerance in Arabidopsis

**Figure 4:**
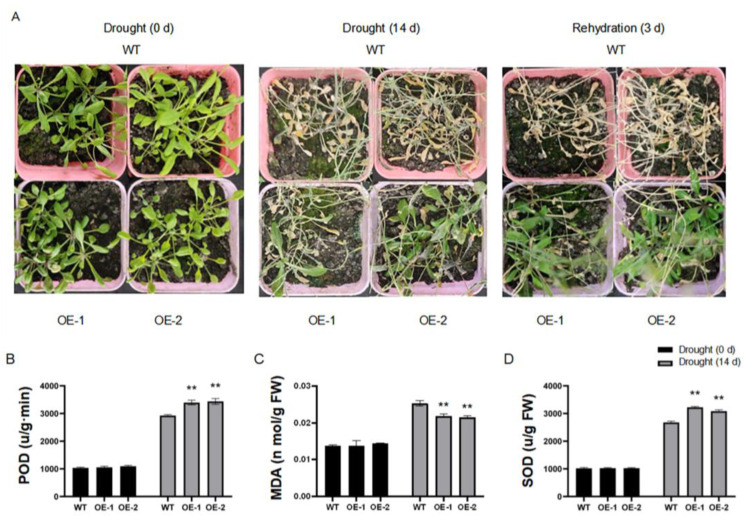
Overexpression of *ZmCML3* enhances the drought tolerance of *Arabidopsis*. (**A**) The phenotype of *ZmCML3* overexpressing lines (OE) and wide type (WT) lines after drought stress treatment for 14 d and rehydration for 3 d. Physiological indexes of the *ZmCML3* overexpressing and WT lines after drought stress treatment for 14 d. (**B**) POD activity, (**C**) MDA content, (**D**) SOD activity. The experiments were performed with three biological replicates. The data were the average (±SD) of three independent experiments. The significance analysis was performed by two-way ANOVA (** *p* < 0.01). For drought stress assay, each biological replicate has no less than 10 plants.

To further explore the function of *ZmCML3*, T3 generation transgenic *Arabidopsis* were obtained and two *ZmCML3* overexpressing *Arabidopsis* lines with the highest expression levels (OE1 and OE2) were used to perform a drought stress assay (Figure S1). The *ZmCML3* overexpressing and wide type (WT) lines were subjected to drought stress treatment by limiting water for 14 d and rehydration for 3 d. As shown in Figure 4A, there were no differences between WT and *ZmCML3* overexpressing lines at 0 d. However, after drought stress treatment for 14 d, the leaves of WT lines showed more severe wilting than those of *ZmCML3* overexpressing lines. After rehydration for 3 d, the growth of *ZmCML3* overexpressing lines was significantly better than that of WT lines. In order to determine the physiological function of *ZmCML3* in response to drought stress, we further measured related physiological parameters. The results showed that there is no significant difference between *ZmCML3* overexpressing lines and WT lines in malondialdehyde (MDA) content and the activity of superoxide dismutase (SOD) and peroxidase (POD) at 0 d. After drought stress treatment for 14 d, the MDA content of *ZmCML3* overexpressing lines was significantly lower than that of WT lines, and the activity of SOD and POD in *ZmCML3* overexpressing lines were significantly higher than that of WT lines (Figure 4B–D).

**Figure 5:**
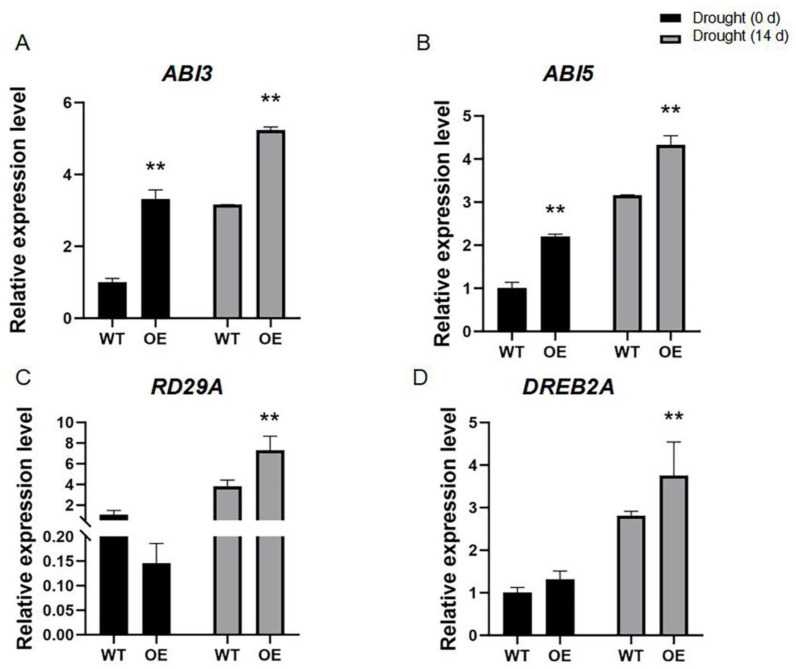
Relative expression levels of drought-stress-responsive genes in *ZmCML3* overexpressing lines and WT lines after drought stress treatment for 14 d. (**A**) *ABI3.* (**B**) *ABI5.* (**C**) *RD29A.* (**D**) *DREB2A*. The experiments were performed with three biological replicates. The data were the average (±SD) of three independent experiments. *ACTIN2* (At3g18780) was used as an endogenous reference for data standardization. Two-way ANOVA was used to perform significance analysis (** *p* < 0.01).

In addition, we investigated the transcription levels of drought stress-responsive genes, such as *ABI3* (AT3G24650), *ABI5* (AT2G36270), *RD29A* (AT5G52310), and *DREB2A* (AT5G05410), which are involved in regulating drought stress tolerance [39,40]. The results showed that the expression levels of these drought-stress-responsive genes in *ZmCML3* overexpressing lines were higher than that of in WT lines (Figure 5A–D). These results indicated that overexpression of *ZmCML3* can positively regulate the drought tolerance in *Arabidopsis* by enhancing antioxidant enzyme activity and the expression of drought-stress-responsive genes.

### 2.6. Overexpression of ZmCML3 Can Enhance Maize Drought Tolerance

**Figure 6:**
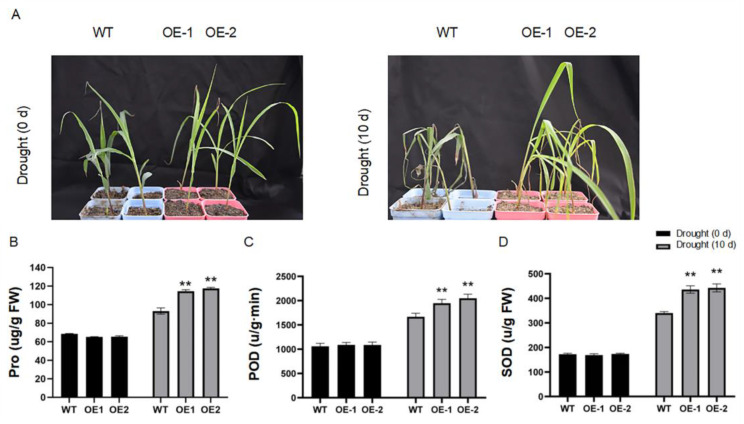
Overexpression of *ZmCML3* enhances the drought tolerance of maize. (**A**) The phenotype of *ZmCML3* overexpressing lines and WT lines after drought stress treatment for 10 d. Physiological indexes in the *ZmCML3* overexpressing and WT lines after drought stress treatment for 10 d. (**B**) Pro content, (**C**) POD activity, (**D**) SOD activity. The experiments were performed with three biological replicates. The data were the average (±SD) of three independent experiments. Two-way ANOVA was used to perform significance analysis (** *p* < 0.01). For drought stress assay, each biological replicate has no less than 10 plants.

In order to further explore the function of *ZmCML3* in maize, T3 generation transgenic maize was obtained and the two overexpressing transgenic lines with the highest expression were selected for further study (Figure S2). The WT and *ZmCML3* overexpressing transgenic seedlings were subjected to drought stress treatment for 10 d at the three-leaf stage. At 0 d, there were no differences between WT and *ZmCML3* overexpressing lines. However, after drought stress treatment for 10 d, the leaves of WT lines showed more severe wilting and curling than those of *ZmCML3* overexpressing lines (Figure 6A). We also measured related physiological parameters after drought stress treatment for 10 d. The results showed that the Proline (Pro) content and the activity of SOD and POD in *ZmCML3* overexpressing lines were significantly higher than that of WT lines (Figure 4B–D).

**Figure 7:**
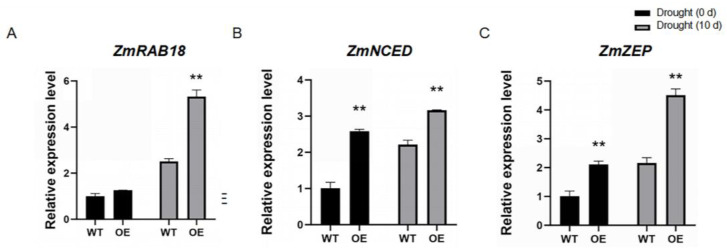
Relative expression of drought-stress-responsive genes in *ZmCML3* overexpressing lines and WT lines after drought stress treatment for 10 d. (**A**) *ZmRAB18*. (**B**) *ZmNCED*. (**C**) *ZmZEP*. The experiments were performed with three biological replicates. The data were the average (±SD) of three independent experiments. *ZmTub* (GRMZM2G066191) was used as an endogenous reference for data standardization. Two-way ANOVA was used to perform significance analysis (** *p* < 0.01).

In addition, we also analyzed the expression levels of drought-stress-responsive genes *ZmRAB18* (GRMZM2G098750)*, ZmNCED* (GRMZM5G838285), *ZmZEP* (GRMZM2G379053), which were involved in maize drought stress response [41]. After drought stress treatment for 10 d, the expression levels of these genes in *ZmCML3* overexpressed lines were significantly higher than those in the WT lines (Figure 7). These results indicated that the *ZmCML3* gene can positively regulate the drought tolerance in maize through increasing the antioxidant enzyme activity and the expression of drought-stress-responsive genes.

## 3. Discussion

Breeding drought-tolerant varieties is of great significance to improve maize yield and protect national food security. To date, many *CML* genes have been found to be involved in plant stress responses. However, the functions of *CML* genes in maize in response to drought stress are unclear. In this study, we cloned a novel *CML* gene in maize, namely *ZmCML3*. ZmCML3 encodes 158 amino acids with three typical EF-hand domains and is located on chromosome 5 (Figure 1B). ZmCML3 showed high identities with Sobic.001G094400.1 (78%) (Figure 1A). ZmCML3 is located within the nucleus and plasma membrane (Figure 3). The expression of the *ZmCML3* gene is significantly induced by ABA, MeJA, NaCl, and PEG6000 (20%) treatments (Figure 2). *ZmCML3* can positively regulate *Arabidopsis* and maize drought tolerance through increasing antioxidant enzyme activity and drought stress responsive gene expression.

The EF-hand motif is the conserved domain in Ca^2+^ sensor proteins, which has an important impact on the function of CMLs. Previous studies have shown that CMLs in *alfalfa* and *barley* contain 1–4 EF-hand motifs [22,42]. *Arabidopsis* CMLs typically have 2–6 EF-hand motifs [26]. CmCML13 and SlCML44 contain three EF-hand motifs, and AtCML15, AtCML16, MsCML10, CpCML15, and gaCaLP contain four EF-hand motifs, respectively [43,44,45,46,47,48]. In this study, ZmCML3 was predicted to contain three EF-hand motifs (Figure 1B). The number of EF-hand motifs varies in different plant species [49,50]. These results indicated that different numbers of EF-hand motifs may play different roles in response to various stresses. Extensive studies have shown that the subcellular distribution of Ca^2+^ sensors may have an important effect on Ca^2+^ signal transduction [11]. An example of this could be drought-tolerance-related genes; CmCML13 was localized to the nucleus, cell membrane, vacuole membrane, and cytoplasmic strand [43]. TaCAM2-D was localized to the nucleus, cell membrane, and cytoplasm [51]. ZoCDPK1 was located in the nucleus and cytosol under normal conditions, but was located in the nucleus and plasma membrane under drought and salt stress treatments [52]. Our research showed that the ZmCML3 is located in the nucleus and plasma membrane (Figure 3). These results suggested that the subcellular distribution of ZmCML3 may play a key role in regulating the signaling pathway in response to drought stress.

Several studies have shown that the *CML* genes participate in drought stress response. Transgenic plants overexpressing *OsCML16* display promoted root growth and improved drought tolerance [53,54]. *CsCML38* was significantly induced under drought stress and ABA treatment [55]. *AtCML9* mutation *Arabidopsis* plants exhibit less wilting after drought stress treatment [56]. After PEG treatment, the expression levels of *TaCML17*, *TaCML30*, *TaCML50*, and *TaCML75* were significantly increased [23]. In wheat, *TaCML20* was significantly induced under water deficit [57]. In this study, *ZmCML3* was significantly induced by drought stress. Meanwhile, *ZmCML3* overexpressing *Arabidopsis* and maize showed better growth than that of WT under drought stress (Figure 4A and Figure 6A). Thus, *ZmCML3* acts as a positive regulator to drought stress, which may play an important role in breeding drought-tolerant varieties. Meanwhile, the expression level of *ZmCML3* is induced by NaCl treatment, indicating that *ZmCML3* may modulate the maize NaCl stress tolerance. The function of *ZmCML3* under NaCl stress needs to be further elucidated.

Increasing studies have shown that plants can improve drought tolerance by increasing the accumulation of compatible solute and antioxidant enzyme activity and reducing the accumulation of ROS [44]. Overexpression of *OsCML4* leads to increased drought tolerance by clearing ROS and inducing stress-related genes [58]. *MsCML46* overexpressing transgenic tobacco displays improved drought tolerance by increasing antioxidant enzyme activity and decreasing ROS production [59]. Similarly to previous studies, the MDA content in *ZmCML3* overexpressing lines were significantly lower than that of WT lines, the content of Pro and the activity of SOD and POD were significantly higher than that of WT lines after drought stress treatment (Figure 4B–D and Figure 6B–D). These results suggested that *ZmCML3* may positively regulate drought tolerance by modulating the antioxidant system and enhancing ROS clearance.

The *CML* genes are involved in response to drought stress via regulating the expression of drought stress-responsive genes. Overexpression of *OsMSR2* (encodes a calmodulin-like protein) enhanced drought tolerance in *Arabidopsis* by altering the expression of stress/ABA responsive genes [60]. In the *AtCML20* mutants, the transcription levels of stress-responsive genes were significantly up-regulated under drought stress [61]. In this study, the stress-responsive genes in *ZmCML3* overexpressing lines were significantly up-regulated (Figure 5A–D and Figure 7A–C). These results indicated that *ZmCML3* can improve drought tolerance by regulating the expression of these drought stress-related genes; however, the detailed function needs to be further studied.

CMLs can modulate drought stress tolerance by activating appropriate signaling pathways [43]. Overexpression of *OsDSR-1* (encodes a calmodulin-like protein) in rice positively regulates the drought tolerance by increasing ABA sensitivity [62]. The drought tolerance gene *PbCML13* could be induced by ABA and MeJA treatments [20]. The drought tolerance related gene *ShCML44* was rapidly and strongly expressed after MeJA treatment [44]. In this study, the *ZmCML3* gene was significantly expressed by exposure to ABA and MeJA (Figure 2). Meanwhile, promoter element analysis showed that *ZmCML3* contains an ABA responsive element (ABRE) (Table S1). These results indicate that *ZmCML3* may be involved in the ABA and MeJA signaling pathways. The genes mentioned above are summarized and listed in Table S3.

## 4. Materials and Methods

### 4.1. Plant Materials and Growth Condition

The seeds of the maize inbred line B73 were grown in a 28 °C/25 °C (16 h light/8 h dark) greenhouse. The three-leaf stage seedlings were treated with ABA (200 µmol/L) [63], MeJA (100 µmol/L) [64], NaCl (200 mmol/L), and PEG6000 (20%) [65], respectively. The leaves were collected at different time points (0 h, 2 h, 6 h, 12 h, 24 h, 48 h, 72 h) and stored at −80 °C.

### 4.2. Isolation and Bioinformatic Analysis of ZmCML3

The full-length CDS of *ZmCML3* was cloned from maize inbred line B73 using Reverse Transcription PCR (RT-PCR). The primers are listed in Table S2. Multiple sequence alignment and homology analysis of ZmCML3 were performed using DNAMAN6.0 software, and the evolutionary tree was constructed using MEGA 5.0 software. The molecular weight of ZmCML3 was analyzed using the Expasy (http://web.Expasy.org/protparam/, accessed on 16 July 2024).

### 4.3. Subcellular Localization of ZmCML3

The CDS of *ZmCML3* was cloned into the GFP-expressing vector pCAMBIA1302 (BgL II and Spe I sites) to construct the ZmCML3-GFP fusion plasmid. The pCAMBIA1302 and ZmCML3-GFP plasmids were transferred into EHA105 *Agrobacterium*, respectively. The recombinant plasmid was transformed into *N. benthamiana* leaves using *Agrobacterium*-mediated method. The *N. benthamiana* leaves were cultured in the dark at room temperature for 72 h and observed via a confocal laser scanning microscope (Leica, Frankfurt, Germany). The primers are listed in Table S2.

### 4.4. Quantitative Real-Time PCR (qRT-PCR) Assay

Total RNA was extracted from the leaves of maize inbred line B73 using Trizol reagent (Tiangen, Beijing, China), and cDNA was obtained using a reverse transcription kit (TOYOBO, Shanghai, China). Quantitative real-time PCR (qRT-PCR) was performed using SYBR Green Master Mix (Genstar, Shenzhen, China) by QuantStudio 3 (Thermo, Waltham, MA, USA). The house-keeping genes, *ZmTub* (GRMZM2G066191) and *ACTIN2* (At3g18780), were used as an endogenous reference [36,62] (Figure S3). The relative expression levels were calculated by the 2^−ΔΔCt^ method with three biological replicates. The primers are listed in Table S2.

### 4.5. Generation of Transgenic Plants

The CDS of *ZmCML3* was ligated to the pCAMBIA3301-CaMV35s expression vector (Nco I and Pml I sites) to construct the pCAMBIA3301-35s-ZmCML3 fusion plasmid. The recombinant expression vector plasmid pCAMBIA3301-35s-ZmCML3 was transferred into *Arabidopsis* (Columbia) by an *Agrobacterium*-mediated floral dip method [66,67]. The transgenic seeds were collected and nurtured in 1/2 MS medium. The seedlings were transferred to vermiculite and cultured in the growth chamber. The seedlings were further screened with 0.001% glyphosate ammonium. T3 generation transgenic *Arabidopsis* were confirmed by Bar gene strip and qRT-PCR. Two *ZmCML3* overexpressing *Arabidopsis* lines with the highest expression levels (OE1 and OE2) were used to perform subsequent experiments. The primers are listed in Table S2.

The CDS of *ZmCML3* was ligated to the pCAMBIA3301-UBI expression vector (BamH I and Mlu I sites) to construct the pCAMBIA3301-UBI-ZmCML3 fusion plasmid. The recombinant vector plasmid pCAMBIA3301-UBI-ZmCML3 was introduced into maize inbred line H99 by an *Agrobacterium*-mediated germinating embryo method [68]. The transformed seeds were planted in the field and screened with 0.01% glufosinate ammonium. Transgenic maize lines were identified using bar test strip and qRT-PCR. Two T3 generation transgenic maize lines with the highest expression levels were used to perform subsequent experiments. The primers are listed in Table S2.

### 4.6. Drought Stress Treatment

T3 generation *ZmCML3* overexpressing *Arabidopsis* (OE) and wild-type *Arabidopsis* (WT) were nurtured on 1/2 MS medium at 23 °C under 16 h light/8 h dark for 7 d. The seedlings were transferred to a planting box and cultured in the growth chamber for two weeks. The seedlings cultured in the planting box were placed in a box and water was absorbed until the soil moisture content reached 80% field capacity. The seedlings were subjected to drought stress treatment by withholding water for 14 d until the soil moisture content reached 30% field capacity, and rehydration for 3 d until the soil moisture content reached 80% field capacity. The *ZmCML3* overexpressing maize lines (OE1 and OE2) and WT were cultured in planting box at 28 °C under light (16 h)/dark (8 h). The three leaf stage maize seedlings were placed in a box and water was absorbed until the soil moisture content reached 80% field capacity. The seedlings were subjected to drought stress treatment by withholding water for 10 d until the soil moisture content reached 35% field capacity. The experiments were performed with three biological replicates, and each biological replicate has no less than 10 plants. The seedlings were pictured using a Nikon D7000 (Nikon, Tokyo, Japan).

### 4.7. Measurement of Physiological Parameters

To further understand the effect of *ZmCML3* on physiological function, we measured physiological parameters, including MDA and Pro content, and the activities of POD and SOD enzymes. These physiological parameters were measured according to the methods described previously [65,69].

### 4.8. Statistical Analysis

One-way ANOVA and two-way ANOVA were used to perform significance analyses. Three biological replicates were performed for each experiment. The data were the average (±SD) of three independent experiments. The data analysis was performed using GraphPad Prism 10 software.

## 5. Conclusions

In this study, we cloned a *CML* gene, namely *ZmCML3*. ZmCML3 was localized within the cell nucleus and plasma membrane. *ZmCML3* can respond to drought and NaCl treatments and its expression is included in the ABA- and MeJA-signaling pathways. *ZmCML3* positively regulates the tolerance to drought stress in *Arabidopsis* and maize through modulating the expression of drought-stress-responsive genes and antioxidant enzyme activity. These results will lay a foundation for revealing the function of *ZmCML3* in maize and provide a reference for breeding drought-tolerant maize varieties.

## Data Availability

All data generated or analyzed during this study are included in this published article and its Supplementary Materials files.

## Supplementary Materials

The following supporting information can be downloaded at: https://www.mdpi.com/article/10.3390/ijms26031329/s1.
