# Supplementary material for: A Maize Calmodulin-like 3 Gene Positively Regulates Drought Tolerance in Maize and Arabidopsis
Source: Int J Mol Sci. 2025 Feb 4;26(3):1329. doi: 10.3390/ijms26031329 (PMC11818628; doi:10.3390/ijms26031329)
Supplement: Supplementary file 1 [file ijms-26-01329-s001.zip › Supplementary Materials Figure S1-S3.pdf]

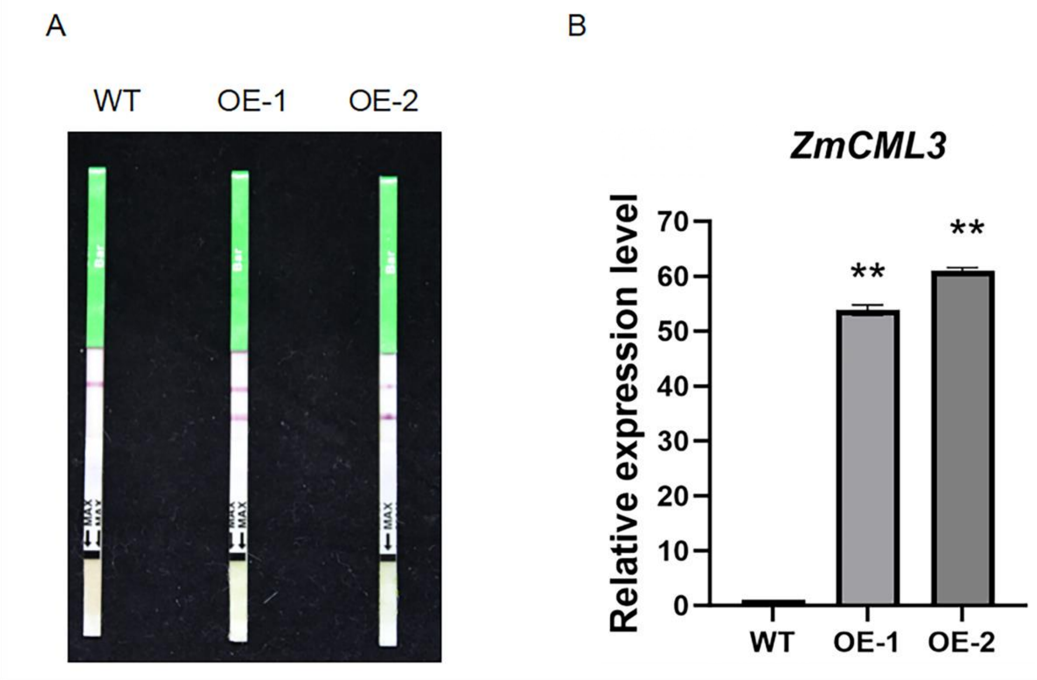

Figure S1. Identification of transgenic *Arabidopsis*. (**A**) Transgenic *Arabidopsis* was detected by bar test strip (**B**) The expression level of *ZmCML3* in overexpressed plants. The experiments were performed with three biological repeats. The data were the average (±SD) of three independent experiments. Two-way ANOVA was used for significance analysis (** $p < 0.01$).

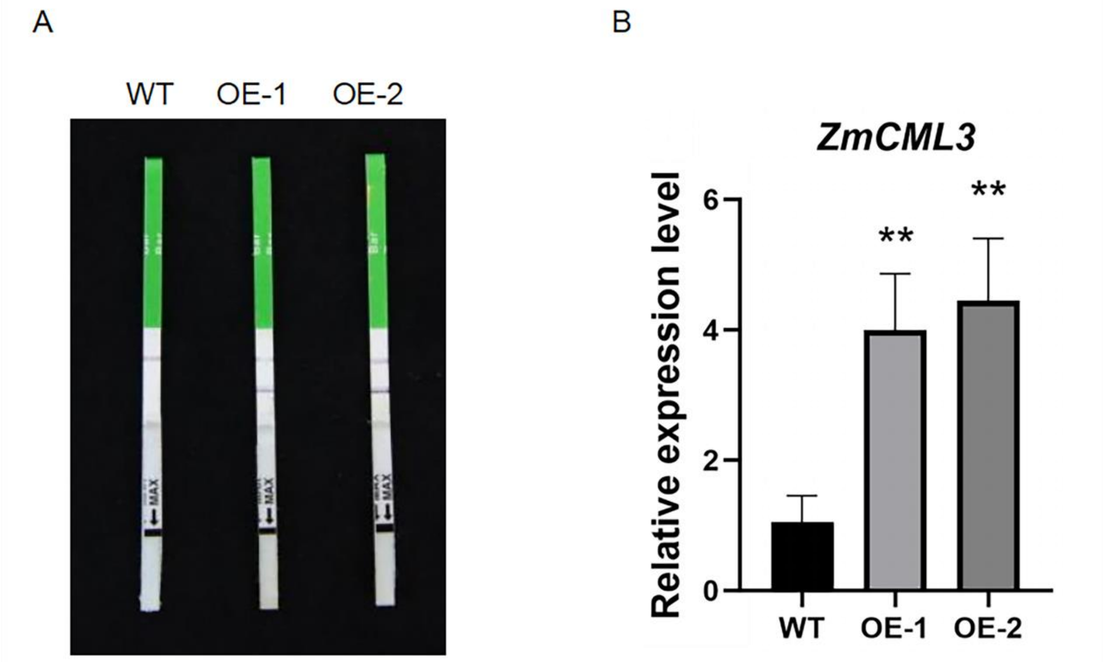

Figure S2. Identification of transgenic maize. (**A**) Transgenic maize was detected by bar test strip. (**B**) The expression level of *ZmCML3* in overexpressed plants. The experiments were performed with three biological repeats. The data were the average (±SD) of three independent experiments. Two-way ANOVA was used for significance analysis (** $p < 0.01$).

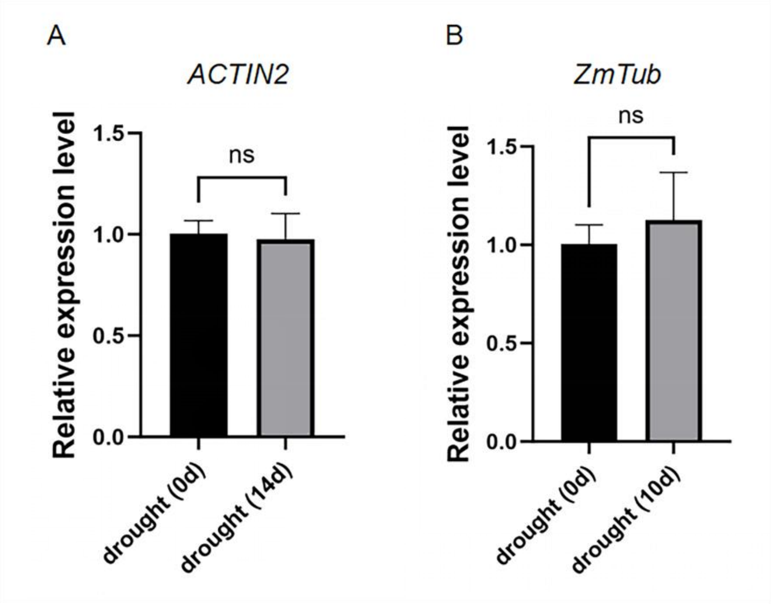

Figure S3. Relative expression level of housekeeping gene under drought stress treatment. (**A**) Relative expression level of *ACTIN2* (At3g18780) in wide-type *Arabidopsis* under drought stress for 14 d. (**B**) Relative expression level of *ZmTub* (GRMZM2G066191) in wide-type maize under drought stress for 10 d. The experiments were performed with three biological replicates. The data were the average (±SD) of three independent experiments. T-tests was used to perform significance analysis (** $p < 0.01$).
